# Mitochondrial Complex I Molecular Alterations in *Sapajus apella* as a Human Gastric Carcinogenesis Model After MNU Exposure

**DOI:** 10.1111/jmp.70017

**Published:** 2025-04-01

**Authors:** Bárbara dos Santos Dias, Symara Rodrigues Antunes, Danilo do Rosário Pinheiro, Rommel Mario Rodriguez Burbano, Bárbara do Nascimento Borges

**Affiliations:** ^1^ Albert Einstein Research and Educational Institute Hospital Israelita Albert Einstein São Paulo São Paulo estado Brazil; ^2^ Metropolitan University Center of the Amazon Belém Pará Brazil; ^3^ Campus Parauapebas Federal Rural University of Amazon Parauapebas Pará Brazil; ^4^ Molecular Biology Laboratory Ophir Loyola Hospital Belém Pará Brazil; ^5^ Cellular Biology Laboratory, Institute of Biological Sciences Federal University of Pará Belém Pará Brazil; ^6^ Molecular Biology Laboratory, Institute of Biological Sciences Federal University of Pará Belém Pará Brazil

**Keywords:** animal model, gastric cancer, mtDNA

## Abstract

**Introduction:**

Gastric cancer (GC) remains among the top five global health problems. Therefore, comprehending the tumor energetic behavior is critical to understanding its progression. This study aimed to investigate mitochondrial DNA (mtDNA) alterations in GC cancer cell lines in an animal model.

**Material and Methods:**

Four mitochondrial genes (*COI*, *ATP8*, *ND1*, and *ND3*) were analyzed in GC (AGP01, ACP02, ACP03, and PG100) and control (Walker 256 carcinosarcoma) cell lines inoculated in *Sapajus apella* , exposed and not exposed to N‐methyl‐N‐nitrosourea.

**Results:**

Two synonymous alterations were identified in *ND1*. In *ND3*, a non‐synonymous alteration (A10398G ➔ Thr114Ala) may decrease the respiratory chain Complex I efficiency, enhancing cellular reactive oxygen species and contributing to mtDNA damage. As alterations in *ND1* and *ND3* were observed in highly aggressive cell lines, our results suggest these genes may play crucial roles in energetic efficiency and gastric carcinogenesis.

## 1 Introduction

Although the occurrence of gastric cancer (GC) has decreased, it still ranks as the fifth‐leading cancer and the fourth‐leading cause of cancer‐related death worldwide [1, 2]. Approximately 90% of diagnosed GCs are adenocarcinomas [3], which can be histologically classified into two types: intestinal and diffuse [4]. The intestinal subtype typically arises from a sequence of pre‐neoplastic lesions (i.e., acute gastritis, chronic gastritis, atrophic gastritis, metaplasia, and dysplasia) [5, 6] and is identified by gland‐like structures. In contrast, the diffuse subtype is characterized by poorly differentiated cells [2]. Gastric Cancer is a global health challenge, often diagnosed late [7], resulting in poor prognosis. Patients with advanced disease face a 5‐year survival rate of 5.3% [8]. Thus, understanding the tumor dynamics is essential to provide new insights into the carcinogenic process and discover new biomarkers.

As a multifactorial complex disease, GC pathogenesis involves nonmodifiable factors, such as age and sex [9], as well as modifiable factors, such as diet, smoking, and *Helicobacter pylori* infection [3, 6, 10]. Notably, excessive consumption of salt, smoked foods, nitrates, nitrites, and poorly preserved foods, associated with the inflammatory environment caused by *H. pylori* infection, alters the gastric microbiota, resulting in increased activity of nitrate and nitrite reductase, allowing the production of carcinogens such as N‐nitrous compounds [11, 12, 13, 14]. These carcinogenic substances may induce mutations in both nuclear and mitochondrial DNAs, resulting in an abnormal formation of Reactive Oxygen Species (ROS) from the oxidative phosphorylation process in mitochondria [15, 16].

N‐methyl‐N‐nitrosourea (MNU), a carcinogenic agent, induces the formation of O6‐methylguanine adducts, leading to premutagenic lesions and DNA strand breaks [17, 18]. Given that various species, including humans, are exposed to carcinogenic MNU generated in their alimentary tract, tumorigenesis induced by MNU is an interesting model for studying gastric cancer [19, 20, 21, 22]. This work aimed to assess alterations in the human mitochondrial genome within human gastric cancer cell lines, both treated and untreated with MNU, inoculated in a primate animal as a gastric carcinogenesis model, previously established by our group [23].

## 2 Material and Methods

### 2.1 Samples

Thirteen *Sapajus apella* specimens, around 6–7 years old and 2.7–3.6 kg, were used in this study. These animals were tagged with microchips and individually accommodated at “Centro Nacional de Primatas” (CENP), Pará State, Brazil. The animals were fed a healthy balanced diet, weighed, and subjected to daily health inspections, during which their clinical symptoms were documented.

The procedures for inoculating gastric cancer cell lines were previously described elsewhere [19, 20]. One week before the cell line inoculation, the *S. apella* specimens were immunosuppressed by a single dose of 50 mg/kg of cyclophosphamide. Three gastric cancer cell lines established by our group (ACP02, ACP03, and AGP01) were used in the experiment, along with two other cell lines (gastric cancer PG100 and Walker 256 carcinosarcoma) as positive controls. All cell lines were percutaneously inoculated between the mucosal and submucosal layers of the antral stomach region of the animals. Ultrasonography was utilized to guide the cell line inoculation procedure and to confirm the generation of a gastric tumoral mass after 72 h.

Each of the three tested gastric cancer cell lines was inoculated on animals, and the resulting tumoral mass was surgically excised at two distinct time points: after 7 and 14 days. For each cell line, a corresponding specimen received daily doses of MNU (16 mg/kg) and had the tumoral mass removed after 7 days. Control cell lines were inoculated using the same procedure and posteriorly removed: PG100 after 7 days (with and without MNU treatment) and Walker‐256 after 7 and 14 days (without MNU treatment). All procedures were carried out by veterinarians affiliated with CENP, and the details of animal welfare and steps taken to alleviate suffering were following the recommendations of the Weatherall report, “The use of non‐human primates in research”. This study was approved by the Ethics Committee of Universidade Federal do Pará (PARECER MED002‐10).

### 2.2 DNA Extraction and Polymerase Chain Reaction

The gastric tumoral masses obtained were submitted to DNA extraction using the QIAamp DNA Mini Kit (Qiagen, Mainz, Rheinland‐Pfalz, Germany) and quantified using a NanoDrop 1000 Spectrophotometer v3.7 (ThermoFisher Scientific).

Polymerase chain reaction (PCR) for *COI*, *ATP8*, *ND1*, and *ND3* genes was performed on all samples using the primers and conditions previously described [24, 25] in a final volume of 25 mL containing: 50 ng of template DNA, 10 pM of each primer, 0.20 mM of each dNTP, 2.5 mM MgCl2, and 0.5 U Taq DNA polymerase (ThermoFisher Scientific). The amplified fragments were sequenced using an ABI 3130 automated sequencer (ThermoFisher Scientific), using the BigDye Terminator v3.1 Cycle Sequencing Kit (ThermoFisher Scientific). The obtained sequences were aligned and analyzed using the BioEdit 7.2.6.1 program [26] and Geneious R11 (https://www.geneious.com), using a human mitochondrial DNA reference sequence (NC_012920.1).

## 3 Results

### 3.1 Cell Line Implantation and MNU Exposure in *Sapajus apella*

All cell lines used in the present study, whether treated with MNU or not, were able to develop tumoral mass growth in the animal model. Furthermore, except for PG‐100, which died during the experiment, all inoculated animals developed cancer within 14 days.

### 3.2 mtDNA Molecular Alterations

**FIGURE 1:**
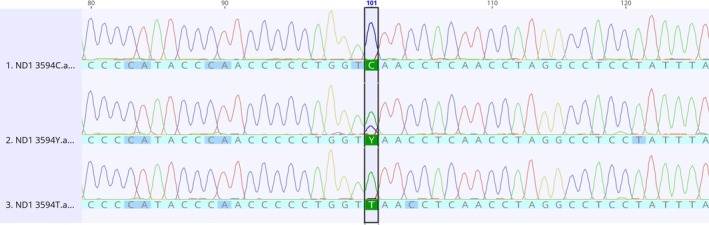
Electropherograms of the *ND1* C3594T alteration. The sequence in the middle shows the heteroplasmic state of the referred alteration.

**FIGURE 2:**
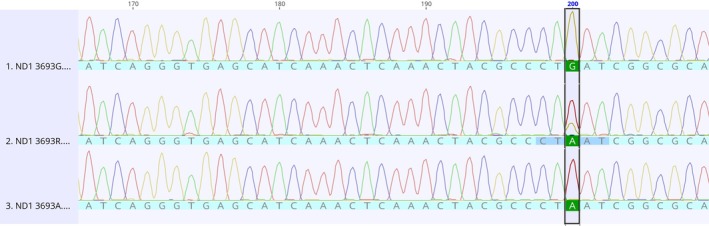
Electropherograms of the *ND1* G3693A alteration. The sequence in the middle shows the heteroplasmic state of the referred alteration.

No DNA changes were detected on the *COI* and *ATP8* genes. On the other hand, two nucleotide changes resulting in synonymous alterations were identified on the *ND1* gene: C3594T transition (Val96Val) (Figure 1), and G3693A (Leu129Leu) (Figure 2). These alterations were observed in the homoplasmic state in ACP03 (14 days and 7 + MNU) and CS256 (7 days and 14 days) and in **the** heteroplasmic state in PG100 (7 days +MNU).

**FIGURE 3:**
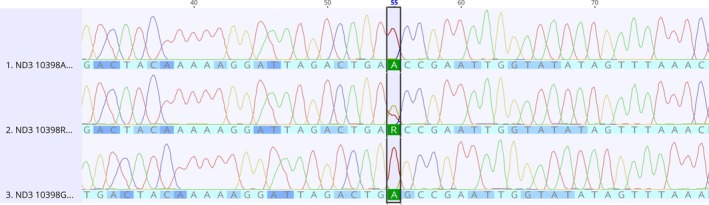
Electropherograms of the *ND3* A10398G alteration. The sequence in the middle shows the heteroplasmic state of the referred alteration.

Regarding the *ND3* gene, an A10398G (Figure 3) non‐synonymous transition (Thr114Ala) was described in ACP03 (14 days and 7 days + MNU), CS256 (7 days and 14 days), and PG100 (7 days +MNU), the latter one observed in a heteroplasmic state.

## 4 Discussion

In addition to being the primary ATP generators in most eukaryotic cells, mitochondria also play important roles in metabolic biosynthesis, as well as in the control of cellular proliferation and apoptosis. As such, they can undergo metabolic reprogramming, especially in several diseases like cancer [27]. In humans, this organelle possesses its own circular, double‐stranded genome, called mitochondrial DNA (mtDNA), with approximately 16 569 base pairs, and encodes 13 protein‐coding genes (responsible for several subunits of respiratory complexes I, III, IV, and V), two ribosomal RNAs, and 22 transfer RNAs [28, 29].

While alterations in the *COI* gene, a Complex III protein‐coding gene, are commonly seen in cancers such as breast [30], prostate [31, 32], and brain tumors [29], our results align with those previously reported [33, 34] in gastric cancer. Furthermore, variations in *ATP8* (a Complex V protein‐coding gene) are rarely described [35, 36]. However, a paired analysis of mitochondrial genomes from eight individuals revealed both germline and somatic variants in *COI* across all samples and somatic variants in *ATP8* in 25% of the samples, suggesting a potential link between these genes and gastric tumor development [37]. Additionally, *COI* alterations were frequently observed in gastric cancer cell lines infected with *H. pylori* [38], indicating potential distinct mitochondrial DNA alteration patterns despite the production of MNU by *H. pylori* [39].

On the other hand, molecular alterations in protein‐coding genes related to Complex I, also known as reduced nicotinamide adenine dinucleotide (NADH)‐ubiquinone oxidoreductase (Q reductase) Complex, are frequent and may significantly impact carcinogenesis. This complex plays a crucial role in cellular responses to oxygen deficiency and the development of hypoxia mechanisms, in addition to resulting in resistance to chemotherapeutic agents that rely on the activation of the redox cycle [28].

Two genes related to Complex I were analyzed in the present study: *ND1* and *ND3*. Regarding the *ND1* gene, the C3594T variation, known as the major SNP defining the L haplogroup (of African origin) [40], has been previously reported in gastric [37] and thyroid tumors [41], suggesting its potential role in cancer development. It is important to note that synonymous mutations, despite not altering the amino acid sequence, can still impact carcinogenesis by influencing protein biosynthesis, folding, and structure [42, 43], potentially acting as driver mutations in cancer development [44].

With respect to the non‐synonymous A10398G, it impacts the *ND3* subunit which is part of the carboxy‐terminal end of respiratory chain complex I [45], resulting in a reduction of the protein aliphatic index [46], decreasing the efficiency of Complex I. It is known that Complex I is the first step in the electron transport chain of the mitochondrial oxidative phosphorylation system, responsible for accepting electrons from NADH and transferring them to ubiquinone [47]. Furthermore, this genetic change enhances cellular ROS production and oxidative stress, potentially contributing to mitochondrial DNA (mtDNA) damage, affecting critical pathways such as the electron transport chain and apoptosis [48, 49], which can initiate and promote tumorigenesis [50, 51]. It is suggested that Complex I dysfunction not only leads to a slightly increased ATP production compared with Complex III alterations but also modestly increases ROS production, promoting enhanced proliferation, survival, invasion, and metastasis capacity of cancer cells [48]. This mutation has been shown to enhance the invasion and metastasis of human breast cancer cells in a mouse xenograft model [52].It has been identified in gastric cancer [37] as well as in several tumor types, such as breast [46, 49, 53, 54, 55, 56], esophageal [56], cervix [57], NSCLC [58], and brain cancer [50].

Heteroplasmy is known as mtDNA variants that co‐exist with the wild‐type mtDNA molecules and can result in alterations in mitochondrial metabolic and energetic functions in the affected tissues. This can influence the severity of diseases as it masks the pathogenicity of heteroplasmic mutations, permitting penetrance only when a certain threshold of heteroplasmy level is exceeded [27, 59, 60].

As for the heteroplasmic state of *ND1* and *ND3* alterations observed only in PG100 cell line, although limited information exists about the original tumor, it is hypothesized that such molecular condition could result from a higher cumulative mutation frequency observed in more aggressive cell lines [61]. On the other hand, it is known that the ACP03 cell line was isolated from a more aggressive intestinal gastric tumor (T4N1M0) compared to the tumors from which AGP01 (T3N2M1) and ACP02 (T3N2M0) cell lines were derived [62]. Interestingly, the 10398G variation was also identified in the control cell line, Walker Carcinosarcoma 256, which exhibits highly aggressive behavior, fast growth, and a short latent period [63]. In ACP03, this variation was observed only after 14 days of implantation in a xenograft model, suggesting an increase in aggressiveness over time, likely attributed to alterations in Complex I. Similarly, the chemical mutagen N‐methyl‐N‐nitrosourea (MNU) appears to enhance strain aggressiveness, as evidenced by the presence of the 10398G variation in both ACP03‐MNU and PG100‐MNU, even with shorter implantation times (7 days).

## 5 Conclusion

Our findings indicate that alterations in protein‐coding genes of the Complex I mitochondrial respiratory chain, particularly *ND1* and *ND3*, are more common in gastric carcinoma cell lines, particularly in highly aggressive ones such as CS256 and ACP03 with and without MNU treatment. These observations suggest that these genes may play crucial roles in gastric carcinogenesis.

## Conflicts of Interest

The authors declare no conflicts of interest.

## Data Availability

The data that support the findings of this study are available from the corresponding author upon reasonable request.
